# Polymeric Nanostructures Containing Proteins and Peptides for Pharmaceutical Applications

**DOI:** 10.3390/polym14040777

**Published:** 2022-02-16

**Authors:** Antiopi Vardaxi, Martha Kafetzi, Stergios Pispas

**Affiliations:** Theoretical and Physical Chemistry Institute, National Hellenic Research Foundation, 48 Vassileos Constantinou Avenue, 11635 Athens, Greece; ada.vardaxi@gmail.com (A.V.); mkafetzi91@gmail.com (M.K.)

**Keywords:** polymeric nanostructures, therapeutic proteins, protein delivery, amphiphilic block copolymers, polyelectrolytes, nanocarriers

## Abstract

Over the last three decades, proteins and peptides have attracted great interest as drugs of choice for combating a broad spectrum of diseases, including diabetes mellitus, cancer, and infectious and neurological diseases. However, the delivery of therapeutic proteins to target sites should take into account the obstacles and limitations related to their intrinsic sensitivity to different environmental conditions, fragile tertiary structures, and short half-life. Polymeric nanostructures have emerged as competent vehicles for protein delivery, as they are multifunctional and can be tailored according to their peculiarities. Thus, the enhanced bioavailability and biocompatibility, the adjustable control of physicochemical features, and the colloidal stability of polymer-based nanostructures further enable either the embedding or conjugation of hydrophobic or hydrophilic bioactive molecules, which are some of the features of paramount importance that they possess and which contribute to their selection as vehicles. The present review aims to discuss the prevalent nanostructures composed of block copolymers from the viewpoint of efficient protein hospitality and administration, as well as the up-to-date scientific publications and anticipated applications of polymeric nanovehicles containing proteins and peptides.

## 1. Introduction

Since human insulin was approved by the FDA (Food and Drug Administration) in 1982, when it became the first commercially available recombinant therapeutic protein [1], several therapeutic proteins and peptides have been endorsed for clinical use and others are under development for the therapy of various diseases such as cancer [2], hepatitis [3], and diabetes [4,5]. Currently, 40% of the 6000 biopharmaceutical products that are in advanced stages of clinical development are protein-based, indicating the prevalence of these products in the future [6].

**Scheme 1:**
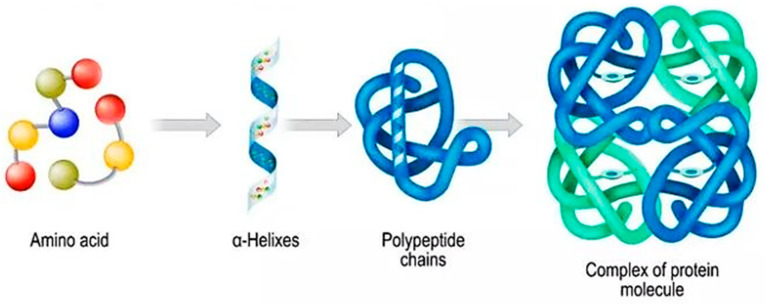
Structural classification of proteins [10].

Proteins and peptides are the dominant biological macromolecules of life that perform essential biochemical functions inside cells, such as enzyme catalysis and signal transduction, as well as being involved in many pathological conditions, such as diabetes and cancer [7]. Cancer onset occurs with the mutation of one or more genes, which causes either the formation of an aberrant protein or hinders the generation of a protein. Irregular proteins deliver dissimilar information to ordinary proteins. This provokes the intractable multiplication of cells, which eventually become cancerous [8]. Both proteins and peptides are comprised of amino acid units and are held together by peptide bonds. Generally, proteins consist of 50 or more amino acids, while peptides are distinguished from proteins based on their size and structure, because they are made up of between 2 and 50 amino acids. Furthermore, peptides are divided into oligopeptides and polypeptides, with the first one consisting of 2–20 amino acids and the last one consisting of 50 or more amino acids [9]. The structure of a protein can be classified into four different forms, namely the primary, secondary, tertiary, and quaternary forms, while the entire three-dimensional structure is dependent on the folding of polyamino acid chains (Scheme 1). The primary structure is related to the sequence of 20 common amino acids located in the protein structure, whereas the more complex structures resulting from the folding and interactions between amino acids are the secondary, tertiary, and quaternary forms [7].

Therapeutic proteins and peptides exhibit tremendous advantages compared to conventional small-molecule drugs. They have advanced specificity and better activity; thus, they cannot be simulated by other chemical compounds. Additionally, they possess less toxicity, which is associated with an immune response during administration [9,11]. However, the high molecular weight of proteins and peptides in conjunction with their varying surface charges and fragile tertiary structures contribute to their limitations in their use as drug molecules, as the capability of their stand-alone delivery into the intracellular space is limited [12]. Furthermore, their high hydrophilicity and the presence of charged groups subsequently lead to poor cell membrane permeability. Moreover, proteins and peptides are susceptible to different environmental conditions, including ionic strength and pH or temperature variations, resulting in the alteration of their physical stability. For instance, their charged groups are vulnerable to the surrounding water molecules because they can interact with them and produce hydrogen bonds, while the peptide bonds in their structure can be influenced by the proteolytic enzymes, which cause proteolytic hydrolysis. Additionally, the restricted permeability across gastrointestinal mucosa, the degradation by enzymes in the gastrointestinal tract (GIT), the reticuloendothelial system (RES) clearance, and the elimination during first-pass clearance effects are obstacles of paramount importance during either oral or parenteral administration of therapeutic proteins and peptides, thereby limiting their clinical application [7,12,13,14,15]. Therefore, several strategies, including the coating of proteins with a protective polymeric layer or their delivery through vehicles at the nanosize scale, are being investigated in order to overcome the aforementioned limitations [16,17].

Regarding the complexity of therapeutic protein and peptide delivery, the field of nanotechnology has effectively bridged the gap, as it provides several nanoscale systems that have the required encapsulating properties, further offering a better pharmacokinetic profile [18,19]. Such nanosystems include lipid nanoparticles [20], liposomes [21], polymeric nanoparticles [22], and magnetic nanoparticles [23], among others. To date, polymeric nanocarriers have gained significant interest as protein delivery vehicles thanks to their multifunctionality. Thus, the enhanced biocompatibility and bioavailability enable these structures to be compatible with living tissues without causing toxic effects in the host, while at the same time protecting the cargo from extreme environmental conditions, subsequently permitting targeted delivery [12,24]. Block copolymers have been extensively utilized in nanostructure construction owing to their structural adaptability, chemical composition, and flexibility. Moreover, the variable molecular weight (Mw) and tunable chemical properties can be tailored to the requirements of each protein–block copolymer [25]. Furthermore, the amphiphilic block copolymers (AmBCs), which exhibit the ubiquitous feature of self-assembly in selective solvents, form structures such as micelles or polymersomes that are eligible vehicles for protein delivery [26,27]. Lastly, polyelectrolytes, amphiphilic block copolymers (AmBCs) comprising at least one polyelectrolyte block, and surfactants belong to an interesting field of macromolecules for clinical use [28].

In this review, the polymeric nanostructures that are utilized for the delivery of therapeutic peptides and proteins and their up-to-date clinical applications are discussed. The design approaches for polymer–protein complexes as well as their connectivity during this process are also analyzed. Eligible architectures such as micelles, nanogels, and polymeric vehicles composed of block copolymers and containing proteins, such as insulin (INS) and bovine serum albumin (BSA) or lysozyme (LYS), are extensively described. Finally, in vitro, in vivo, and current pharmaceutical applications over the last five years are also reported. 

## 2. Polymeric Nanostructures 

Polymeric materials have opened new horizons in protein and drug delivery; hence, they have been adopted as subjects of great interest from scientists working in these fields. Their high biodegradability, the adjustable control of both the structure and chemical arrangement, their colloidal stability, and the potential for integration of a plethora of either hydrophobic or hydrophilic molecules are a few of the promising properties that they possess [29,30]. Nevertheless, it is necessary for these nanostructures not to trigger cytotoxicity causing toxic impacts to healthy tissues, but to provide proper host response and extended therapeutic effects [31]. Moreover, these novel multifunctional nanoplatforms can contribute to diagnosis and therapy (theranostics) in a single system [32,33].

Polymeric nanostructures are formed through the self-assembly process of short polymer chains or more complicated polymer architectures, such as dendrimers and multibranched polymers. Stimuli-responsive polymers, so-called smart polymers, are the key factor in the latest generation of supramolecular nanocarriers, which are formed through the combination of the two categories. These polymers are able to undergo physicochemical changes or rearrangements due to their response to a specific stimulus, whether that be endogenous, such as to pH and redox variations, hypoxia, and enzymes, or exogenous, such as temperature alterations, light exposure, magnetic fields, and ultrasound, thereby enabling controlled release of active pharmaceutical ingredients (API) at specific sites [32,34,35].

**Scheme 2:**
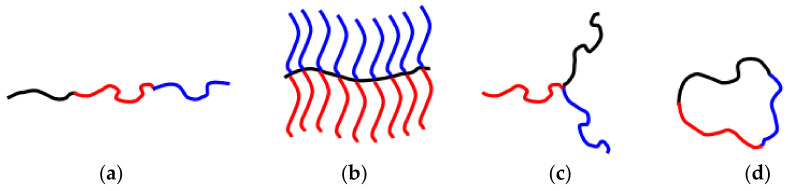
Representative examples of (**a**) linear triblock terpolymer, (**b**) “comb” graft polymers, (**c**) “miktoarm” star terpolymers, and (**d**) cyclic block terpolymers [38].

Block copolymers (BCPs) with precise molecular weights, compositions, and architectures have received massive attention thanks to their ability to form explicit structures at the nanoscale that can be used as nanocarriers of biomolecules [36]. They are comprised of two, three, or more distinct blocks of diverse chemical groups, joined together with covalent bonds, leading to the construction of diblock, triblock, or multiblock copolymers, respectively. Each block is composed of the repetition of a single monomeric unit. Furthermore, BCPs are classified along with their structural array to linear, star-shaped, graft, branched, and cyclic architectures, whereas their ultimate configuration arises from the regulation of parameters in the synthetic process (Scheme 2). The Flory–Huggins interaction parameter, the polymerization degree, the volume fraction, the molecular weight, and the proportion of each component block result in the differential demonstration of nanomorphologies, e.g., spherical, cylindrical, lamellar [37,38].

Living or controlled radical polymerization (CRP) strategies are the most facile and multifaceted path for BC preparation. They are congruent with an abundance of monomers. Furthermore, they exhibit sufficient acceptance of functional groups. Three main controlled radical polymerization (CRP) methodologies have been reported regarding the preparation of BCs, including atom transfer radical polymerization (ATRP), reversible addition–fragmentation chain transfer (RAFT) radical polymerization, and nitroxide-mediated polymerization (NMP) [39].

The RAFT approach has been established as the most advantageous method for acquiring well-defined BCs, as it is an easily manipulable method that is compatible with a wide range of monomers that bear functional segments. In addition, the RAFT protocol allows control over molecular characteristics such as the molecular weight and composition through the proper adaptation of the polymerization conditions. The diverse molecular architectures, absence of catalysts, possible realization of the polymerization process in aqueous media, soothing experimental conditions, high precision over the final product with well-appointed side-groups, and post-polymerization functionalization are only a few of the beneficial features that RAFT polymerization provides [39,40,41].

Block copolymers can be further classified according to the nature of the constituent blocks and their affinity with water. AmBCs consisting of at least one hydrophobic and one hydrophilic block self-assemble in aqueous solutions, further forming micellar-like nanostructures or nanoaggregates. The self-assembly process of a block copolymer results in the formation of a corona–shell-type micelle, whereby the hydrophobic block comprises the core of the nanoparticle and the hydrophilic block comprises the shell that surrounds the core and prevents the contact of the hydrophobic part with water molecules [5,26,42]. During the self-assembly process, electrostatic interactions, hydrogen bonding, π–π aromatic stacking, and van der Waals forces altogether influence the organization of macromolecules, while by tailoring the experimental parameters, the preferable structures can be collected. This procedure also promotes reduced interfacial free energy, which arises from the need to decrease the interfacial area of non-soluble parts. Moreover, according to solvophilic and solvophobic segments of AmBCs, distinguishable morphologies such as micelles, lamellae, and vesicles can be observed. Remarkably, AmBCs micelles contribute to the delivery of solubilized water-insoluble molecules such as proteins, as reviewed in the following section [37,43]. Furthermore, the amenability of molecular features of the self-assembly process provides the ability of different polymers to be combined. Self-assemblies of mixtures of random and block copolymers can be tuned to distribute their hydrophobic parts in a controlled way, taking advantage of the statistical distribution and covalent connection along the polymer chains. However, these blended polymeric coassemblies require further investigation in terms of the effectively distinct release of drugs and proteins [42,44,45]. 

Among the AmBC-based nanoassemblies, the ones obtained via the self-association of neutral copolymers are influenced by the thermodynamic equilibrium, whereas polyelectrolyte block copolymers are controlled by the strong electrostatic interactions and the release of counterions [46]. Polyelectrolytes have ionizable units, either acidic or basic, which accept or donate protons, respectively, and respond to exposure to different stimuli. In other words, polyelectrolytes can be considered as polymers constituted from repeating ionizable units. Moreover, the self-assembled polyelectrolyte block copolymers integrate the key characteristics of BCs, polyelectrolytes, and surfactants; hence, the ease of structural modification for these intriguing classes of macromolecules allows various possibilities for utilization as nanostructural delivery vehicles of drugs, proteins, peptides, and genes. These responsive nanoassemblies can deliver the therapeutic agents through electrostatic interactions, as discussed comprehensively in the following chapter [28,46,47,48].

## 3. Linking Polymers, Proteins, and Peptides towards the Formation of Polymer–Protein–Peptide Nanostructures

### 3.1. Polymer–Protein Conjugation

**Scheme 3:**
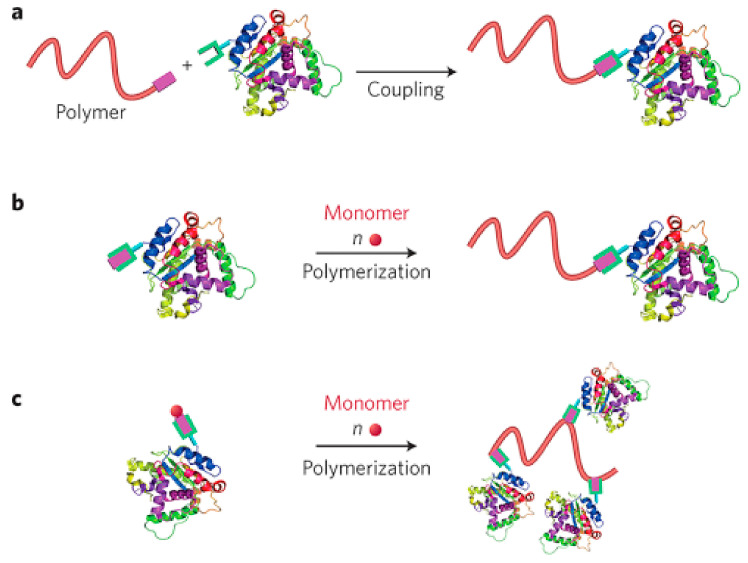
(**a**) Grafting to, (**b**) Grafting from, and (**c**) grafting through methods used for preparing protein–polymer bioconjugates. Reprinted with permission from Ref. [52]. Copyright 2018, American Chemical Society.

Protein–polymer bioconjugation comprises an approach that results in hybrid biomacromolecules that combine both the efficient and morphological features of synthetic polymers and natural proteins [49]. The process of polymer–protein conjugation is accomplished via three different, the “grafting to”, “grafting from”, and “grafting through” strategies (Scheme 3). The “grafting to” method is based on the anchorage of polymers to proteins through coupling interactions, while the polymer moiety is produced separately and prior to the final step of hybrid molecule formation. However, the low yield of the reactions resulting from the steric hindrance among these macromolecules limits its further utilization. Moving on, the “grafting from” approach was demonstrated when living polymerizations techniques came to the forefront and entails the in situ growth of a polymer chain from the protein or peptide to produce well-defined hybrid architectures. A small-molecule initiator or chain transfer agent (CTA) is primarily amended with the target protein through bioconjugation reactions, subsequently producing a macroinitiator from which controlled polymerization is generated in order for the polymer chain elongation to be completed. Among the living or controlled radical polymerization (CRP) techniques, reversible-addition−fragmentation chain transfer (RAFT) [40] and atom transfer radical polymerization (ATRP) [50] fulfill the requirements in terms of the controllable conjugation site, polydispersity, and chain length, allowing an exceptional yield of the hybrid molecule [51,52]. To conclude, the latter strategy of polymer–protein conjugation is called the “grafting through” strategy and is accomplished either after the synthesis of a macromonomer–protein complex for further polymerization or by the conjugation of multiple proteins to a polymer pursued via the polymerization and formation of a comb-like structure with high density. However, the low polymerization degree and complicated nature of the final product restricted the usage of this approach [52]. 

### 3.2. Chemical Bonding of Polymer Chains with Protein

Due to the increasing interest in polymer–protein conjugates, scientists have benefited from the properties of specific polymers and applied them for protein stabilization or functionalization. Since the first covalent bonding of poly(ethylene glycol) (PEG) to bovine serum albumin (BSA) introduced by Abuchowski and colleagues in 1997 [53], numerous publications have reported on the conjugation of polymer chains to proteins. Proteins are fragile molecules that are able to be denatured when exposed to different environments and have a low half-life in blood and tissues. Hence, the chemical bonding with polymer chains increases the hydrodynamic volume while offering improved solubility, better physiological stability, and reduced immunogenicity [13,52]. The covalent conjugation with PEG polymers, also known as PEGylation, is one of the widespread strategies used for protein guarding and stabilization [54,55]. To date, over 10 PEGylated therapeutic proteins have been approved by the FDA, including Adagen^®^, Somavert^®^, Oncaspar^®^, and Naloxego^®^, which have been introduced to the pharmaceutical market, whilst other products are in clinical development [56]. Although the effective stabilization of therapeutic proteins is achieved during PEGylation, the addition of PEG chains diminishes their binding affinity and bioactivity. Zwitterionic polymers, which are polyelectrolytes characterized by equal positive and negative groups on the chains and an overall neutral charge, can be applied to overcome the weaknesses of PEG conjugation [57]. Moreover, instead of linear chains of PEG, branched or graft-like polymers such as poly(oligo(ethylene glycol) methyl ether methacrylate (POEGMA) could be alternatively utilized to improve the pharmacokinetics of therapeutic proteins. The methacrylate part in the monomer enables the site-specific polymer conjugates to be achieved using the “grafting from” strategy, while the side ethylene glycol chains demonstrate stealth properties [58].

On the contrary to the covalent-based bonding of polymer–protein bioconjugates, which generates powerful and stable connections between them, the non-covalent strategy produces architectures with superior specificity and reversibility [52]. Non-covalent approaches to biocomplexation rely on interactions such as electrostatic interactions, hydrophobic interactions, hydrogen bonding, and protein–polymer coordination, yet this linking is prone to different stimuli, such as salt, temperature, and pH; thus, the environmental alterations may influence the core–corona structures of such polymeric nanoparticles, leading to their dissociation [59]. Additionally, studies have proposed non-covalent PEGylation as a favorable tailoring approach for managing protein stability. Appropriate candidate for the non-covalent binding of PEG to protein surfaces could be diblock copolymers, including PEGylated polyelectrolytes. The attachment via strong electrostatic interactions prevents protein denaturation [60,61]. 

### 3.3. Protein Encapsulation

Proteins can be loaded into polymeric nanocarriers to provide protection from extreme environmental conditions and to extend their function until they are efficiently released at the target site [62]. Protein encapsulation can be conducted through two approaches, physical embedding and chemical bonding. Hydrophobic connections and electrostatic interactions are involved in physical embedding, whereas chemical linking of PEG (PEGylation) is involved in the chemical bonding process. Apropos of hydrophobic association, therapeutic proteins are encapsulated in the micellar core of amphiphilic block copolymers when micellization occurs [63]. On the other hand, when a block copolymer with a neutral and a charged block is mixed with an oppositely charged protein, polyion complex micelles (PIC) are formed in aqueous media with a narrow size distribution. Electrostatic interactions are mainly responsible for the PIC–protein formations, while hydrogen bonding can sufficiently contribute to the binding of the polymer and protein [64].

## 4. Classes of Polymeric Protein and Peptide Nanocarriers

Proteins including insulin, monoclonal antibodies, growth factors, enzymes, and peptides have revolutionized the pharmaceutical industry due to their high specificity and lower toxicity compared to small conventional drugs. Nevertheless, their vulnerable structure, high Mw, and short half-life are some of the notable limitations that need to be taken into consideration [65,66]. Therefore, polymeric nanocarriers comprised of block copolymers and forming architectures such as micelles, nanogels, nanocapsules, and nanovesicles, among others, have been coordinated with proteins and peptides. These nanoplatforms beneficially amend the features of therapeutic molecules, prevent denaturation, and facilitate the transport and release at the desired site [67]. 

### 4.1. Micelles

**Scheme 4:**
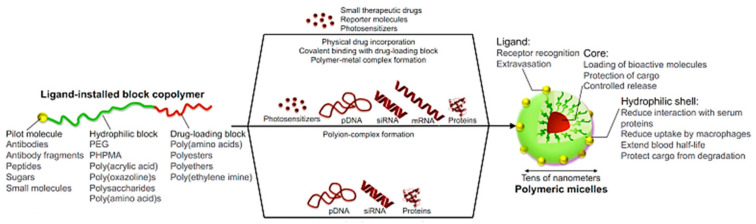
Self-assembled formation of polymeric micelle utilizing ligand-installed block copolymer for encapsulation of therapeutics. Reprinted with permission from Ref. [42]. Copyright 2018, American Chemical Society. Direct link: https://pubs.acs.org/doi/abs/10.1021/acs.chemrev.8b00199, accessed on 28 January 2022.

Micelles are versatile amphiphilic nanostructures created by the self-assembly of block copolymers and have a tremendous impact in the biopharmaceutical field, offering multiple functions and clinical advances. Their size range is from 10 to 100 nm and they are able to be arranged into different morphologies, such as spherical micelles (the widespread form), cylindrical micelles, and lamellae. Different micellar morphologies resulting from the self-assembly of different block copolymer classes are depicted in Scheme 4. Generally, polymer micelles can be divided into three main categories based on the intramolecular forces that control their formation: (1) micelles formed by hydrophobic interactions; (2) micelles resulting from electrostatic interactions; (3) non-covalently bonded polymeric micelles. Owing to the astonishing feature of therapeutic molecule encapsulation into their core, they are qualified vehicles for the delivery of hydrophobic proteins and peptides [68]. Regarding the polymers utilized for the construction of hydrophobic and hydrophilic portions of AmBCs with micellar structures, polyester, polyether, or poly(amino acid) derivatives are chosen for the former, whilst poly(ethylene glycol) (PEG), poly(acrylamide) (PAAm), poly(hydroxyethyl methacrylate), poly(N-vinylpyrrolidone) (PVP), and poly(vinyl alcohol) are preferred for the latter due to their hydrophilicity. Additionally, the ability of some polymers to respond to different stimuli has contributed to the design of thermo-responsive, pH-responsive, enzyme-responsive, and redox-responsive micelles [69]. The hydrophobic polymers act as depots filled with solubilized lipophilic molecules, namely biodegradable polymers that tend to prefer a non-aqueous environment, such as poly(β-benzoyl-L-aspartate), poly(ε-caprolactone) (PCL), poly(D,L-lactic acid), polyethers (i.e., poly(ethylene oxide) (PEO), and poly(propylene oxide) (PPO), protecting them from the surrounding media. On the other hand, the shell or corona of micelles has a crucial role in preventing opsonization and protein degradation. Moreover, it determines fundamental characteristics such as the size, charge, lipophilicity, and hydrophilic block surface density of the micellar structures, as well as communication with the environment. Due to the presence of the hydrophilic shell, there is the capability for the introduction of ligands. It should be noted, however, that the selection of the eligible blocks should not only be correlated with the final micellar nanostructure, but also with their safety. Regarding the FDA regulations and given the fact that multiple administration doses of micelles are required, the selective block copolymers need to be biocompatible, biodegradable, and to have a non-toxic profile in order to safely be excreted from the human body without bringing about accumulation [5,42,70,71].

The key factor correlated with the construction of self-assemble micelles for protein or drug delivery is their stability behavior underneath highly diluted circumstances in body fluids. The stability can be studied through the critical micelle concentration (CMC). When amphiphilic block copolymers are dissolved in a selective solvent beyond a specific concentration, micellization occurs. The CMC of AmBC micelles has been reported in the range of 10^−6^ to 10^−7^ M, which is lower than micelles of low-MW polymers (10^−3^ to 10^−4^ M). The low CMC needed for their production results in their high stability upon dilution and contributes to drug and protein delivery processes, as the active pharmaceutical ingredients loadings are completed at low concentrations [5,26,72,73]. A supplementary factor that also triggers the micellization process is the critical micelle temperature (CMT). Both the CMC and CMT allow thermodynamic parameters, such as the enthalpy of micellization, to be verified [5,68].

#### 4.1.1. Conventional Micelles

The term “conventional micelles” corresponds to micelles that are formed due to the hydrophobic interactions between core–corona regions in aqueous media [74]. Among living or controlled radical polymerization (CRP) techniques, reversible-addition−fragmentation chain transfer (RAFT) and atomic transfer radical polymerization (ATRP) could be utilized for the lengthening of the hydrophobic blocks [75]. Nevertheless, there is a novel “block-copolymer-free” approach for micelles, which relies on the self-assembly of random copolymers, graft copolymers, homopolymers, or oligomers, while interpolymer hydrogen bonding complexation may also be responsible for the micellization process. Due to intramolecular-specific interactions among the core–corona, these micelles are defined as non-covalently bonded structures [68]. Pluronics^®^ or poloxamers are the most well-known studied systems in aqueous solutions due to their similarities with low-MW surfactants that form aggregates and micelles [76]. Likewise, POEGMA is a copolymer with an enhanced reputation due to the presence of hydrophilic ethylene oxide (EO) side groups, which act as stealth moieties with excellent solubility and a low critical solution temperature [76]. Pirs-Oliviera et al. [76] observed the effects of molecular structures on the aggregation of POEGMA random copolymers through an ATRP process utilizing OEGMA monomers of different molecular weights (2, 5, 20, and 45 EO units). Copolymers with micellar-like structures or large phase-separated aggregates can be formed at well-defined temperatures and at low concentrations, allowing the potential use of these nanocarriers for the delivery of hydrophobic molecules.

Andrade et al. [77] suggested the use of amphiphilic triblock copolymers of poly(ethylene oxide)-b-poly(propylene oxide)-b-poly(ethylene oxide) (PEO-PPO-PEO) with the commercial name Pluronics^®^ or poloxamers for the preparation of micellar-like structures. In their work, Soluplus^®^, Pluronic^®^ F68, Pluronic^®^ F108, and Pluronic^®^ F127 were utilized to produce lyophilized micelles for insulin delivery through inhalation. The overall aim was to collect dry powders composed of block copolymer micelles for pulmonary delivery of insulin. Phenylboronic acid (PBA) was also introduced into the nanoformulations due to its glucose-sensitive properties and capability for insulin-controlled release, thereby converting them into stimuli-responsive structures, but it did not eventually provide the desirable glucose-sensitive properties. After the micellization process, micelles were lyophilized in order to obtain solid formulations with high stability. According to the cytotoxicity assays, the lyophilized powders did not present in vitro toxicity to respiratory cells. Among the block copolymers, Pluronic F68 and Pluronic F108 demonstrated rapid and higher release of insulin compared to Soluplus and Pluronic F127, which presented more sustained release, making them the micelles of choice for long-acting powders. In conclusion, micelles based on Pluronic F128 have shown promising properties as nanoplatforms for pulmonary administration of insulin in the future.

Kamenova et. al. [78] designed coassembled mixed micelles combining two different triblock copolymers, poly(2-(dimethylamino)ethyl methacrylate)-b-poly(εcaprolactone)-b-poly(2-(dimethylamino)ethyl methacrylate) (PDMAEMA_20_-b-PCL_70_-b-PDMAEMA_20_) and poly(ethylene oxide)-poly(ε-caprolactone)-b-poly(ethylene oxide) (PEO_113_-b-PCL_35_-b-PEO_11_), and examined their adequacy as nanocarriers for the encapsulation and delivery of insulin. The preparation of PDMAEMA_20_-b-PCL_70_-b-PDMAEMA_20_ was conducted through the ATRP process of PDMAEMA initiated by a PCL initiator and of PEO_113_-b-PCL_35_-b-PEO_11_ through a copper-mediated “click” coupling reaction. The mixed block copolymer micelles arise from the blended triblock copolymers at molar ratios of 7:3, 3:7, and 1:1. After the coassembly into the water, the core consisted of the hydrophobic PCL blocks, while the corona was comprised from two layers, namely the middle corona layer from mixed PEO/PDMAEMA segments and the outer corona layer from PEO. Subsequently, the immobilization of insulin into micelles was achieved through its complexation via electrostatic interactions with the positively charged PDMAEMA blocks. The results indicated that the complexation process, the physicochemical features of mixed micelles, and the in vitro cytotoxicity are affected by the PEO/PDMAEMA ratio. The dominance of PEO reduced the insulin loading efficiency, whilst the excess of PDMAEMA increased the cytotoxicity. Hence, the blended copolymers at a ratio of 1:1 demonstrated the appropriate proportion for fruitful insulin therapy.

Li and coworkers [79] synthesized enzyme-responsive micelles composed of poly(ethylene glycol)-b-poly(2-diisopropylaminoethyl methacrylate) (PEG-b-PDPA) block copolymers to study the insulin release behavior under glucose alterations for the treatment of diabetes mellitus (DM). The well-defined PEG-b-PDPA was produced by ATRP technique using PEG-Br as the initiator, while insulin was fluorescently labeled in order to be easily detected in the sample. When the pH value was 7.4, PEG-b-PDPA self-assembled into micelles in which the core contained the hydrophobic PDPA polymer chain and the corona the hydrophilic PEG chains. Fluorescein-labeled insulin (FITC-insulin) and GOD (glucose oxidase) were colabeled into the polymeric micelle. The controlled MW, the relatively low polydispersity index (PDI = 1.34), and the low CMC demonstrated that the formed micelles had adequate thermodynamic stability in high-dilution environments, indicating that they could be potentially utilized in vivo. Moreover, due to the enzymatic catalysis of glucose and GOD, which further produce gluconic acid, the pH value of the microenvironment is decreased. This subsequently leads to the tertiary amine groups of PDPA being protonated and transitioning from the hydrophobic to the hydrophilic state. The pH-responsive disassembly could beneficially contribute to the rapid release of FITC–insulin in response to a glucose stimulus. The cytotoxicity assays displayed up to 95% of block-copolymers cell viability; thus, the glucose-responsive PEG-b-PDPA micelles are efficient insulin delivery nanocarriers.

#### 4.1.2. Polyion Complex Micelles (PICs)

Polyion complex micelles (PICs) are formed when a negatively charged block copolymer containing a hydrophilic non-ionic block is mixed with a positively charged homopolymer or a cationic-neutral block copolymer, and vice versa, through electrostatic interactions and van der Waals forces. During this approach, the use of an organic solvent is avoided, eliminating the side effects caused by the organic solvent and providing PICs as appropriate candidates for the delivery of therapeutic ingredients, such as nucleic acids and proteins [80]. Besides the efficient delivery of proteins, which is comprehensively illustrated in this review, small interfering RNA (siRNA) and DNA containing PICs have been studied in-depth and are presented in the literature [81]. Hydrophilic or hydrophobic substances and charged molecules can be entrapped into the core of PICs through hydrogen bonding and hydrophobic and electrostatic interactions, while the binding between them arises from the conjunction of the ionic functionality and polymer architecture. Moreover, the strength of electrostatic interactions relates to the aforementioned parameters, as well as to the environmental conditions. In addition, PICs are nanostructures able to assemble and disassemble, and similar to conventional micelles, their formation is characterized by the critical aggregation concentration (cac) [5,64]. Concerning the PIC core, it can either consist of cationic or anionic polymers in accordance with the enclosed therapeutic proteins. Poly(L-lysine)/(P(Lys), poly(aminoalkyl aspartamide) and poly(aminoalkyl methacrylate)/(PAMA) are cationic blocks that interact with positively charged proteins, whereas poly(acrylic acid)/(PAA) and poly(aspartate)/(P(Asp)) interact with negatively charged proteins [64].

**Scheme 5:**
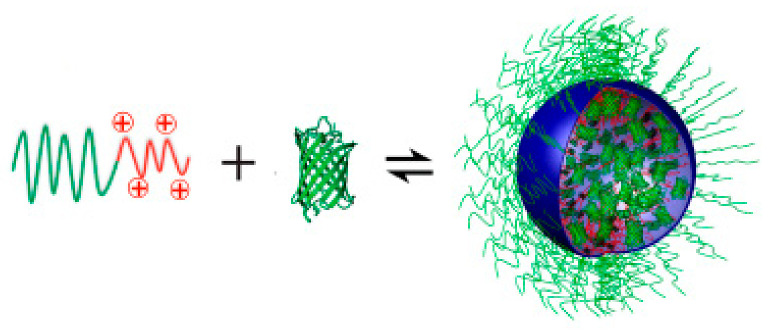
Graphical representation of protein–block polyelectrolyte micelle formation utilizing green fluorescent protein. Reprinted with permission from Ref. [85]. Copyright 2015, American Chemical Society.

Polymers that contain ionizable units are defined as polyelectrolytes. Given the fact that PICs originate from the coassembly of oppositely charged polymers, they can also be described as polyelectrolyte complex micelles (PECs). When a diblock copolymer containing a neutral, hydrophilic block and a charged polyelectrolyte block is mixed with an oppositely charged protein, PECs are collected through microphase separation, in which the core consists of protein-charged blocks and the corona consists of neutral blocks [28,82]. A depiction of the protein–block polyelectrolyte micelle formation is shown in Scheme 5. Pippa et al. [83] studied the electrostatic complexation among a cationic-neutral block copolymer and a protein. The polyelectrolyte was the poly(quaternized bis(dimethylamino hydroxy styrene)-b-poly(ethylene oxide) (QNPHOS-b-PEO) copolymer, in which the PEO blocks provided stealth properties to the nanostructure and insulin was utilized as the negatively charged protein. The parameters such as the structure, size distribution, ζ-potential, and solution stability depend on the ratio of the two components, the pH, as well as the ionic strength during the complexation process. In conclusion, since the initial demonstration of self-assembled PECs was reported by Kataoka and Harada in 1995 [84], in which the oppositely charged block copolymers poly(ethylene glycol)–block-poly(L-lysine) (PEG–b-P(Lys)] and poly(ethylene glycol)–block-poly(α,β-aspartic acid) [PEG–b-P(Asp)) were mixed, a plethora of studies on PECs have been conducted. Their fundamental properties and applications as nanovehicles for the delivery of biologically active substances such as proteins and peptides have been demonstrated.

Jiang et al. [86] attached maleimide-functionalized poly(oligo (ethylene glycol) methyl ether methacrylate) (MI-POEGMEMA) to BSA in order for this BSA conjugate to be used for the production of polyelectrolyte complex micelles with lysozyme (LYS) or with the endogenous angiogenesis inhibitor Sprouty-1. The synthesis of MI-POEGMEMA was performed through RAFT polymerization and a furan-protected maleimide-chain transfer agent. The cytotoxicity assays were implemented against breast carcinoma cell lines (MDA-MB-231 and MCF-7), which indicated that POEGMEMA-BSA conjugates with a 1:1 molar ratio were not toxic. The results showed that the negatively charged BSA and unstable positive charged Sprouty-1 effectively formed stable micelles due to the presence of a water-soluble POEGMA block, while at the same time the delivery of the inhibitor was facilitated. These novel polyelectrolyte–protein complex micelles can potentially be investigated for use with other cationic proteins against cancer.

Wang and colleagues [87] aimed to produce a biocompatible nanoformulation composed of cationic CA-PLGA-b-(PEI-PEG) micelles for insulin or other protein delivery. In particular, star-shaped poly(D, L-lactide-co-glycolide)/(PLGA) functionalized by cholic acid and the crosslinked low-MW polyethyleneimine and polyethylene glycol were self-assembled into cationic micelles, while they complexed with insulin through electrostatic interactions at pH = 7.4. The corona of PEI-PEG offered a strong positive charge to the micelles, while the core of CA-PLGA provided hydrophobicity. Subsequently, Wang et al. studied the in vitro release profile of insulin when it was encapsulated into microspheres (INS-MS) and through micelle–insulin-complex-loaded CA-PLGA microspheres (MIC-MS). The subsequent in vitro experiments demonstrated that the cationic micelles improved the release of insulin from MIC-MS while the animal studies in diabetic rats showed sustained and prolonged hypoglycemic effects using MIC-MS.

Another important contribution to the field of protein delivery by polymeric vectors was reported by Chen and coworkers [88]. They prepared concavo-convex micelles with ameliorated bioactivity and stability as a consequence of the self-organization of poly(methyl methacrylate)-b-poly(sodium p-styrene sulfonate) (PMMA-b-PSS)–shellac enzyme bioconjugates. The group revealed that the bioactivity and stability of the loaded enzyme block copolymer micelles hit high rates of 300% and 760% respectively compared to the native shellac enzyme, whilst the unloaded micelles presented bioactivity of 208% and stability of 622%. The noteworthy action of the complexes is assigned to the amended contact potential between the polymeric substrate and the shellac enzyme. The peculiar polymer–shellac enzyme complexes are efficacious for degrading catechol with increased efficacy compared to the original enzyme. The aforesaid process proposed by Chen and his group describing the preparation of concavo-convex nanoparticulates will promote their employment for environmental maintenance and catalytic enhancement.

Skandalis et al. [89] prepared QPDMAEMA-b-PLMA-b-POEGMA cationic amphiphilic triblock terpolymer micelles as nanocarriers for insulin encapsulation. The synthesis of poly(quaternized 2-(dimethylamino)ethyl methacrylate-b-lauryl methacrylate-b-(oligo ethylene glycol)methacrylate) was accomplished by sequential RAFT polymerization, while the quaternization reaction of PDMAEMA with methyl iodine converted the tertiary amine groups into quaternary amine groups with permanent positive charges. The amine and quaternary ammonium terpolymers self-assembled into micelles with PLMA cores and (Q)PDMAEMA-POEGMA mixed coronas, which further complexed with insulin electrostatic interactions, as insulin is negatively charged at pH = 7. The physicochemical studies demonstrated the QPDMAEMA-b-PLMA-b-POEGMA/insulin complexes were colloidally stable under all insulin concentrations utilized, whereas insulin conformation was not affected by the complexation process with the cationic triblock terpolymer micelles, indicating that these nanoformulations can be applied for insulin delivery. 

### 4.2. Hydrogels

Hydrogels are three-dimensional water-soluble networks that are controllably crosslinked through physical or chemical methods. They are mainly composed of hydrophilic and biocompatible polymers, either natural or synthetic, and they display biocompatibility that further provides them the ability to swell in the aqueous media in which they are dispersed. They are widely applied in the pharmaceutical field, in biosensors [90,91], as biomaterials in tissue engineering [92], in cancer therapy [93], or as drug delivery carriers [94]. Specifically, they enable hydrophilic active therapeutic biomolecules to be effectively delivered. To name but a few, 2-hydroxyethyl methacrylate (HEMA), ethylene glycol dimethylacrylate (EGDMA), N-isopropyl acrylamide (NIPAAm), acrylic acid (AA), methacrylic acid (MAA), poly (ethylene glycol) (PEG), and poly (vinyl alcohol) (PVA) are utilized in hydrogel construction for protein delivery. Moreover, the mechanical properties of hydrogels are closely correlated with the degree of hydrogel crosslinking; hence, a stronger structure is connected to a higher crosslinking degree that sometimes needs to be avoided when hydrogels are intended for protein delivery [95,96]. In addition, they can be designed in order to undergo sol–gel transition, dissolution, or degradation due to environmental alterations. These hydrogels are defined as “intelligent” or stimuli-responsive networks able to alter their network swelling behavior or physicochemical characteristics under exposure to different stimuli, such as pH, temperature, or ionic strength. Particularly, pH-triggered hydrogels tailor their structural behavior to different pH-related conditions. Thus, ionic hydrogels protect proteins from the harsh gastric fluids and are swollen when exposed to the alkaline environment of the intestine, enabling their sustained release [95,97].

**Scheme 6:**
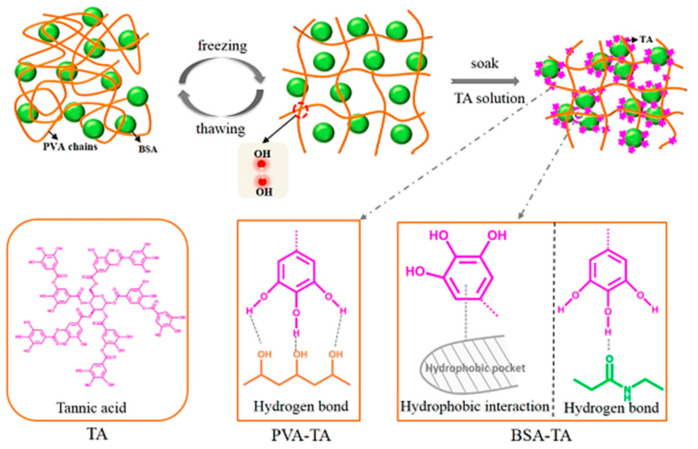
Schematic illustration of the development of tannic acid–PVA/BSA hydrogels via hydrogen bonding and hydrophobic interactions. Reprinted with permission from Ref. [99]. Copyright 2018, American Chemical Society.

Regarding the existing literature, three main hydrogel–protein delivery systems have been identified. Firstly, the conjugation of hydrogels with proteins through covalent (chemical) crosslinks produces hydrogels with enhanced stability, in which the polymer–protein ratio has an effect on gel formation, while the protein release is dependent on the rate of polymer degradation [96]. Recently, Phan et al. [98] synthesized a triblock copolymer consisting of poly(ε-caprolactone-*co*-lactide)-*b*-poly(ethylene glycol)-*b*-poly(ε-caprolactone-*co*-lactide (PCLA) and BSA. The chemically crosslinking was improved due to the protein complexity, forming hydrogels capable of accelerating the wound healing and tissue regeneration without using additional inorganic nanoparticles. Furthermore, in the physically entangled hydrogel–protein systems produced by weak intermolecular interactions, the proteins are slowly disintegrated with the simultaneous polymer dissolution [96]. Xu et al. [99] synthesized a promising double-protein–hydrogel network with high convergence and water resistance. Firstly, a PVA solution was integrated with BSA, forming the primary hydrogel, then tannic acid (TA) was crosslinked through hydrogen bonding and hydrophobic interactions with the BSA/PVA network, with the aim of mechanically improving the pure PVA hydrogel. The result was the production of a TA-BSA/PVA double-pure network with superior tensile strength, which is adjustable and dependent on the TA/PVA ratio and soaking time in TA. A schematic depiction of the investigation conducted by Xu et al. [99] is shown in Scheme 6.

Hydrogels can also be swelled after contact with water, allowing the diffusion of proteins from the pores on their structure. The degree of swelling and the size of the pores determine the protein release [97]. Lima and coworkers [100] prepared alginate-based hydrogels as delivery systems, anticipating the efficient delivery and oral release of BSA. The swelling behavior was examined in acidic and basic environments, and as far the results showed, the swelling profile was pH-dependent at the higher pH value of 7.4, while at pH = 1.2 alginate–BSA hydrogels reached pseudoequilibrium. In addition, cell viability studies proved the compatibility of Alg-based hydrogels with enhanced pharmacological activity. Similarly, Sabaa et al. [101] encapsulated BSA into xanthan gum-poly (N-vinyl imidazole) (XG-PVI)-based hydrogels, which can be utilized as oral delivery systems. Their aim was to evaluate the kinetic release profile of protein at pH = 7.4. Protein loading (%) and encapsulation efficiency (EE%) values showed direct correlations with the gelation time and BSA concentration and inversely with the polymer concentration. The XG-PVI-BSA hydrogel exhibited a high loading efficiency, excellent biocompatibility, and no cytotoxic effects. In conclusion, the release profile of BSA in both experimental works was related to the polymer concentration, while the successful cytotoxic assays indicated that alginate and xanthan gum are probably appropriate for oral protein delivery.

Polymers bearing acidic or basic functional groups tailor their behavior in accordance with their responses to different environmental stimuli. These smart polymers are proposed for protein delivery, particularly insulin, in the human body. Hydrogels based on polycationic polymers preserve the release of insulin in the acidic environment of the stomach (pH = 1.0–3.0), while they shrink in the intestine due to the high pH (pH~7.4) level, which causes neutralization of positive groups, enabling the release of protein. On the other hand, hydrogels composed of polyanionic polymers are in neutral status in the stomach, but in the alkaline environment of the intestine they swell, releasing loaded insulin as a consequence of the negative charge of the polymer groups [97]. Li et al. [102] produced a copolymeric pH-responsive semi-interpenetrating hydrogel with improved physicochemical properties, biocompatibility, and non-toxic profile for insulin oral delivery. The polymer network was composed of poly(vinyl alcohol)/poly(hydroxypropyl methacrylate-co-methacrylic acid) (PVA/P(HPMA-co-MAA)) and was produced through free-radical polymerization of HPMA and MAA in the presence of PVA. SEM characterization exhibited the presence of pores located in the structure, which afterward contributed to the hydrogel’s excellent swelling traits under different conditions. The in vitro studies indicated that these pH-responsive hydrogels protected the insulin in the stomach compared to the intestine, where the release was beneficially increased. Interestingly, in the oral administration of insulin in diabetic rats, blood glucose levels were reduced, indicating that these copolymer pH-responsive hydrogels could potentially be applied as vehicles for oral insulin administration. An alternative nanogel for oral insulin delivery was proposed by Wang et. al. [103]. Hydroxyethyl methacrylate (HEMA) was utilized due to its low fouling nature, which provides the ability for blood circulation to be increased, provoking more efficient absorption of protein in the intestine. The hypoglycemic effects of diabetic rats and the bioavailability were found to last longer compared to oral delivery of free insulin.

Moreover, injectable hydrogels comprised of biodegradable stimuli-responsive copolymers have gained attention because they are able to undergo the sol–gel transition during injection. Sim et al. [104] produced anionic injectable hydrogels of Hep-PCLA/heparin-poly(ε-caprolactone-co-lactide)-b-poly(ethylene glycol)-b-poly(ε-caprolactone-co-lactide) for the release of positively charged lysozyme. PCLA is a stimuli-responsive polymer, as it responds to temperature variations in aqueous solutions, enabling the formation of the hydrogel at human body temperature (37 °C). The result of sol-to-gel state changes at room temperature (25 °C) was that the anionic polymer had a sol phase while the solution was rapidly formed into in situ gel after the subcutaneous injection into the backs of rats. The lysozyme-based Hep-PCLA-injectable hydrogels demonstrated decreased in vivo initial burst, good biocompatibility, and enhanced sustained release of lysozymes. Likewise, Turabee et al. [105] developed pH- and temperature-responsive polypeptide-based injectable hydrogels for positively charged protein administration. The anionic pentablock copolymer OSM-b-PBLG-b-PEG-b-PBLG-b-OSM is composed of pH-responsive oligo(sulfamethazine), hydrophilic poly(ethylene glycol), and temperature-responsive poly(γ-benzyl-L-glutamate). The polypeptide copolymer exhibited a sol phase at room temperature, while it acquired the gel state at 37 °C, after the subcutaneous injection into rats. Similarly to previous research, lysozyme-polypeptide-based injectable hydrogels displayed controlled sustained release and a safe profile.

Li and coworkers [63] published an equally significant study on novel bifunctional, bio-decomposable nanogels able to load and release insulin according to predetermined glycose levels. Poly(N-isopropylacrylamide) (PNIPAM) nanogels were prepared with nitrilotriacetic acid (NTA) and phenylboronic acid (PBA) as active groups, while ethylene glycol dimethacrylate (EGDMA) acted as the crosslinker. Specifically, the NTA segments bind with insulin through chelation or absorption of zinc ions into the nanogels, prompting adequate insulin loading. Imaging studies showed well-constructed nanostructures of spherical morphology. The installation of PBA triggered the nanogels with glucose alertness. The loading capacity of the encapsulated insulin into the NTA-chelated Zn(II) nanogels climbed to 66%. Glucose-sensitive and regulated release of insulin was accomplished. Finally, cytotoxicity and enzymatic decay assays of the nanogels disclosed both sufficient biodegradability and biocompatibility.

### 4.3. Polymeric Vesicles

Amphiphilic block copolymers are also capable of self-assembling into nanocapsules, vesicles, or polymersomes. The aforementioned distinct nanostructures are of significant interest for the distribution of proteins and genes, as they can successfully encapsulate them inside the inner aqueous compartment and release them at the site of action.

#### 4.3.1. Polymersomes 

Polymeric vesicles described as “polymersomes” are spherical hollow vesicles with a polymeric lamellar structure as a membrane. The aqueous core is surrounded by a bilayer membrane exposing hydrophilic coronas. Active biomolecules with relatively small sizes such as proteins are able either to be covalently attached towards the outer surface of polymersomes or encapsulated into the liquid core [106]. Moreover, these vesicles can encapsulate hydrophilic molecules or integrate them, establishing them as popular delivery systems. Nevertheless, the physicochemical parameters and the ratio of hydrophilic–hydrophobic sections for the block copolymer construction should be tailored to the needs of the final vesicular structures. For instance, the overall polymersome size defines the circulation time of the nanostructure, as well as its bio-distribution and clearance. However, the factors that influence the size and size distribution of polymersomes are not well comprehended. Furthermore, the proportions of hydrophilic and hydrophobic blocks in the block copolymers affect the final polymersome structure, as has also been demonstrated in micelles, where the copolymer is mainly hydrophilic. The inherent curvature, which arises from the balance among soluble and insoluble parts, is low, with a packing parameter of 1/2 ≤ *p* ≤ 1. Additionally, nanosized polymersomes are produced when the polymer concentration is high, whereas microsized polymersomes are collected in mild conditions [26,47].

**Scheme 7:**
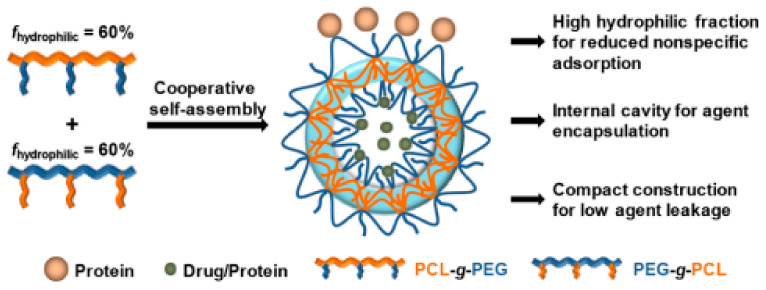
Graphical representation of compact vesicle preparation composed of binary graft copolymers with high hydrophilic segment content for drug and protein delivery applications. Reprinted with permission from Ref. [108]. Copyright 2017, American Chemical Society.

Polymersomes are able to encapsulate both hydrophobic and hydrophilic molecules, in comparison to micelles, which encapsulate only hydrophobic ones, and they possess higher stability in circulation. The thin outer membrane (2–47 nm, depending on the length of the block copolymer chain and its composition) results in superior entrapment of hydrophobic molecules, while they are also less agile and more stable [107]. Among the block copolymers utilized for polymersome construction, researchers have proposed that graft copolymers allow better enhancement of protein absorption and controlled release as a consequence of the hydrophilic–hydrophobic balance. Weng and coworkers [108] demonstrated the collaborative self-assembly of binary graft copolymers with reversed hydrophilic–hydrophobic blocks into polymersomes. The binary systems were composed of poly(caprolactone)-g-poly(ethylene glycol) (PCL-gPEG, GCP_o¯i_) and poly(ethylene glycol)-g-poly(ε-caprolactone) (PEG-g-PCL, GCP_i¯o_) and had the same hydrophilic–hydrophobic ratio. The tremendous biocompatibility and blood compatibility when BSA and lysozyme were encapsulated suggest the binary graft copolymers can potentially be used as delivery nanocarriers (Scheme 7).

It has been proven that polymersomes possess high specificity with remarkably low toxicity, thereby indicating the integration of therapeutic proteins, as they also improve their half-life time [109]. Nomani et al. [106] proposed that mPEG-PCL polymersomes could be considered potential carriers for protein release. Biodegradable hydrophobic poly(ε-caprolactone) and biocompatible, hydrophilic methoxypoly(ethylene glycol) blocks self-assembled, producing mPEG-PCL polymersomes through the double-emulsion technique (w/o/w). Regarding the physicochemical characterization, the 1:4 BSA/polymer ratio provided the upper limit of loading and sustained released, as well as the minimum polydispersity index, while the high integration efficiency was provided by the strong BSA–polymer interactions. 

Another widespread application of polymersomes as protein delivery nanocarriers is in transcutaneous patches for insulin delivery. Hu and coworkers [110] produced a closed-loop glucose-responsive insulin platform. The glucose-responsive formulations that incorporated glucose oxidase (GOx) were based on the enzymatic oxidation of glucose to gluconic acid (glucose + O_2_ + H_2_O → gluconic acid + H_2_O_2_, with the presence of GOx). The polymeric vesicles (polymersomes) were H_2_O_2_-sensitive and composed of polyethylene glycol (PEG) and phenylboronic ester (PBE)-conjugated polyserine. PBE was chosen due to its H_2_O_2_-mediated degradation. After the self-assembly of block copolymers, GOx and insulin were encapsulated into the interior of mPEG-b-P(Ser-PBE). The polymeric vesicles subsequently integrated into hyaluronic acid (HA)-based microneedle array patches. When the patch was applied to diabetic mice, the glucose, which was spread out in the membrane because of hyperglycemic effects, interacted with GOx, leading to the oxidation of glucose to gluconic acid and the production of H_2_O_2_. As a consequence, the mPEG-b-P(Ser-PBE) insulin vesicles responded to the presence of H_2_O_2_ and permitted the release of insulin. This pioneering insulin patch could clinically contribute to the emergence of the closed-loop insulin delivery approach, as it exhibits biomimetic behavior similar to the pancreatic beta cells that rapidly release insulin when high glucose levels are detected. Moreover, these nanosystems have the beneficial feature of normoglycemic state detection, which is related to decreased insulin release when glucose levels are at normal rates, thereby avoiding the risk of hypoglycemia.

#### 4.3.2. Nanocapsules

The in situ formation of polymeric coatings around the proteins produces formations known as nanocapsules, which are hollow spherical structures with dimensions at the nanoscale. Nanocapsules are typically empty shells with a hollow inner space composed of polymers and known as “reservoir” systems due to their ability for protein entrapment inside the aqueous core. They are characterized by important features in comparison to traditional polymeric protein or drug delivery nanostructures, including the efficient encapsulation of large cargoes inside their cavity with simultaneous protection by the polymer shell. Moreover, their high surface area allows surface modifications with agents through physical embedding or chemical conjugation. For these reasons, nanocapsules have been investigated as drug delivery systems for biopharmaceutical applications. Remarkably, biodegradable nanocapsules that are stimuli-responsive (pH, redox, enzyme) permit the on-demand release of the encapsulated protein, allowing precise and controlled administration, although their clinical application requires further investigation [111,112].

Briefly, nanocapsules are formed through three strategies, namely nanoprecipitation, nanoemulsion, and the layer-by-layer technique. In nanoprecipitation, alternatively named the interfacial deposition approach, the organic solvent is added via a thin needle into water. Once the nanocapsules are formed, the organic solvent is removed by diffusion or evaporation [113]. Furthermore, the nanoemulsion strategy is based on the emulsion–diffusion–evaporation method of organic solvent production, where the nanocapsules are formed through the combination of polymer precipitation and interfacial assembly during diffusion. Additionally, for the nanoemulsion process, there is an emulsion–coacervation approach in which polyelectrolytes or polymers bearing crosslinking functional groups comprise the shell of nanocapsules, which is subsequently stabilized through coacervation or chemical crosslinking. It is noteworthy that emulsion–coacervation can be combined with RAFT [114] and ATRP [115] for nanocapsule production. Finally, the layer-by-layer method produces multilayer nanocapsules and is based on the sequential deposition of polyanions and polycations on the inorganic core through electrostatic interactions between oppositely charged layers [113]. 

Liang et al. [116] proposed a novel strategy to produce a polymeric nanocapsule that acts as a shield to therapeutic proteins, offering stealth properties, extended circulation times and low immunogenicity. 2-Methacryloyloxyethyl phosphorylcholine (MPC) and N,N′-methylenebisacrylamide (BIS) were utilized as the monomer and crosslinker, respectively, producing a thin layer around the protein through in situ polymerization. The zwitterionic polymer of the shell provides stealth features to the nanocapsule, as well as resistance to non-specific protein attachment, and does not allow protein identification from the cells of the immune system. The technique of in situ polymerization is an easy method of nanocapsule design and production for the encapsulation of therapeutic proteins. 

A very thorough study concerning redox-responsive polymeric nanocapsules loaded with active caspase 3 (CP-3) or BSA and protein distribution along the cytosols of cells was proposed by Zhao and coworkers [117]. The synthetic approach they followed for the preparation of single-protein nanocapsules involved blending the protein with acrylamide (AAm), a positively charged N-(3-aminopropyl) methacrylamide (APMAAm), and a disulfide-containing crosslinker. The monomers were electrostatically adsorbed onto the surface of the protein and in situ polymerization started after the introduction of free radical initiators. In order to trigger reversible crosslinking of the capsule under reducing conditions, cleavable disulfide bonds containing N,N-bis(acryloyl)cystamine were employed. The nanocapsules were demonstrated to be efficiently internalized into cells and to discharge the protein in the reducing cytosol environment. They subsequently triggered apoptosis in a broad spectrum of human cancer cell lines, such as HeLa, MCF-7, and U-87 MG. In summary, the group proposed a redox-responsive encapsulation strategy that is facile yet efficacious for intracellular protein distribution.

## 5. Potential Applications

The interest in the use of proteins and peptides as therapeutic agents to combat diseases such as diabetes or cancer has increased dramatically. Due to the advantages that they possess, polymeric nanostrcutures have been utilized as vehicles intended to protect the therapeutic molecules and to extend their lifetime until their release at the site of action. However, only a few examples of treatments using therapeutic proteins have been successfully translated into clinical applications. Supplementary to the aforementioned up-to-date in vitro and in vivo studies for the prevalent nanostructures, this section will briefly present some potential applications for polymeric nanostructures containing insulin, growth factors, and monoclonal antibodies.

Glucose-responsive systems are well-known approaches for insulin delivery to treat diabetes [118]. Li and coworkers developed a novel, biocompatible, sensitive peptide hydrogel comprising glucose oxidase, catalase, phenyboronic acid (PBA), and glucose-binding proteins. Entrapped insulin was used with the aim of efficiently releasing it from the hydrogel after the detection of glucose concentrations. The choice of peptides as the building blocks was related to the provision of biocompatibility and ligand recognition into the materials that form the final hydrogel. During hypoglycemia, glucose is oxidized to gluconic acid while the local pH is below pKa, leading to the ultimate disassembly of the hydrogel and the release of insulin. The in vivo studies indicated that the hydrogel was injectable and could effectively control blood glucose levels. However, further in vivo studies are required for the appropriate dosage of insulin to be optimized.

The use of polymer-based scaffolds is another approach for the delivery of growth factors, with applications in tissue engineering; however, this approach has had little clinical validation [119]. Kim et al. [120] produced a fibrous poly(D,L-lactide-co-glycolide)/(PLGA) scaffold incorporated with BMP-7 (bone morphogenetic protein-7)-loaded PLGA polymeric nanoparticles for the repair of osteochondral imperfections in rabbits. These systems were subsequently blended with synovium-resident mesenchymal stem cells (synMSCs), providing a fruitful microenvironment for their growth, proliferation, and differentiation. After the transplantation to osteochondral defects of rabbits of scaffold-supported synMSCs, the cartilage regeneration was provoked with improved proteoglycan and collagen type II deposition. To conclude, the intra-articular delivery of both BMP-7 and synMSCs through PLGA scaffolds can be potentially applied in articular cartilage repair.

Monoclonal antibodies (mAds) have emerged as a class of essential therapeutics for the treatment of various diseases, including cancer and central nervous system (CNT) diseases. However, the delivery of therapeutic drugs and proteins into the CNT is extremely restricted due to the blood–brain barrier (BBB). Han et al. [121] designed an effective nanoplatform for mAds delivery to CNT for brain tumor therapy. 2-Methacryloyloxyethyl phosphorylcholine (MPC), composed of choline and acetylcholine analog-based nanocapsules, was synthesized with a peptide crosslinker through polymerization. During this process, a thin polymeric shell around the monoclonal antibodies was developed, thereby producing the nanocapsules. Moreover, the peptide crosslinker was utilized due to the cleavage abilities of metalloproteinase-2 (MMP-2), which is a proteinase that is overexpressed in brain tumors, while the polymers enhance the penetration through the BBB. When the nanocapsule enters the BBB, the proteinase located in tumors is activated due to the peptide presence, leading to the break of the nanocapsule shell and further release of mAds to stop the tumor proliferation. These nanosystems did not exhibit significant impacts on hepatic and renal functions, nor did they produce injury in mucosal systems, indicating they could be potentially used for brain tumor therapy.

## 6. Conclusions

Therapeutic proteins and peptides exhibit a plethora of advantages that cannot be ignored; thus, they have attracted considerable attention for use in combating various chronic diseases such as diabetes, cancer, and neurological or infectious diseases. Nevertheless, their unfavorable inherent limitations, such as their fragile structure, low half-life, and instability when exposed to different stimuli, are obstacles that have led to the development of approaches for their efficient delivery. Polymeric nanostructures, mainly composed of amphiphilic block copolymers and forming architectures such as micelles, hydrogels, and nanovesicles, contribute to the efficient delivery of proteins and peptides. These nanoplatforms beneficially protect the bioactive molecules from enzymatic degradation or denaturation, as well as preventing their release until they reach the site of action. Despite the fact that further studies and stringent clinical validation are required before these pioneering nanostructures are able to sustainably and efficiently deliver therapeutic proteins or peptides for practically utilization, the future of their applications is envisioned to be auspicious.

## Data Availability

Not applicable.
